# Incidence of *Campylobacter concisus* and *C. ureolyticus* in traveler’s diarrhea cases and asymptomatic controls in Nepal and Thailand

**DOI:** 10.1186/s13099-017-0197-6

**Published:** 2017-08-17

**Authors:** Oralak Serichantalergs, Sirigade Ruekit, Prativa Pandey, Sinn Anuras, Carl Mason, Ladaporn Bodhidatta, Brett Swierczewski

**Affiliations:** 1 Department of Enteric Diseases, Armed Forces Research Institute of Medical Sciences, 315/6 Rajvithi Road, Bangkok, 10400 Thailand; 2 CIWEC Clinic Travel Medicine Center, GPO Box 12895, Kapurdhara Marg, Kathmandu, 44600 Nepal; 3 Bumrungrad International Hospital, 33 Soi Sukhumvit 3, Khwaeng Khlong Toei Nuea, Khet Watthana, Bangkok, 10110 Thailand

**Keywords:** *Campylobacter*, Travelers, Diarrhea, Nepal, Thailand

## Abstract

**Background:**

*Campylobacter concisus* and *C. ureolyticus* have emerged in recent years as being associated with acute and prolonged gastroenteritis and implicated in the development of inflammatory bowel diseases. However, there are limited data on the prevalence of these microorganisms in Southeast Asia. In this study, 214 pathogen-negative stool samples after laboratory examination for common enteric pathogens to include *C. jejuni* and *C. coli* by culture from two case–control traveler’s diarrhea (TD) studies conducted in Thailand (cases = 26; controls = 30) and Nepal (cases = 83; controls = 75) respectively were assayed by PCR for the detection of *Campylobacter* 16S *rRNA* and two specific heat shock protein genes specific for *C. concisus* (*cpn60*) and *C. ureolyticus* (*Hsp60*) respectively.

**Results:**

*Campylobacter* 16S *rRNA* was detected in 28.5% (61/214) of the pathogen-negative TD stool samples (CIWEC Travel Medicine Clinic, Kathmandu, Nepal: cases = 36, control = 14; Bamrungrad International Hospital, Bangkok, Thailand: cases = 9, controls = 2). *C. consisus* was identified significantly more often in TD cases in Nepal (28.9%; 24/83) as compared to controls (4%; 3/75) (OR = 9.76; 95% CI 2.80–34.02; *P* = 0.0003) while *C. consisus* was detected in only two cases (2/26; 7.7%) and none of the controls stool samples from Thailand. *C. ureolyticus* was detected in four cases (4.8%; 4/83) and four controls (5.3%; 4/75) and in one case (3.8%; 1/26) and one control (3.1%; 1/30) from Nepal and Thailand respectively. *C. jejuni* and *C. coli* were isolated in 18.3 and 3.4% of the cases and in 4.0 and 1.4% of the controls in stool samples from both Thailand and Nepal respectively while *C. concisus* nor *C. ureolyticus* were not tested for in these samples.

**Conclusion:**

These findings suggest that *C. concisus* potentially is a pathogen associated with TD in Nepal. To our knowledge, this is the first report of *C. concisus* and *C. ureolyticus* detected from traveler’s diarrhea cases from travelers to Nepal and Thailand.

## Background

*Campylobacter* spp. are common causes of human bacterial gastrointestinal disease worldwide in particular *C. jejuni* and *C. coli* are recognized as the leading cause of acute bacterial diarrhea worldwide accounting for nearly 400 million cases per year [1, 2]. *Campylobacter*-associated gastroenteritis is highly prevalent in South and Southeast Asia and is commonly associated with traveler’s diarrhea (TD) [3–6]. Over the last decade, recent evidence of other *Campylobacter* spp. to include *C. ureolyticus* and *C. concisus* has highlighted the potential role of these microorganisms to cause gastroenteritis in both industrialized and developing countries [7, 8].

Several *Campylobacter* spp. (non-*C. jejuni/coli*) were isolated in the early 1990s from diarrhea stool samples though the ability to culture these organisms was highly variable among laboratories [9, 10]. Many *Campylobacter* spp. require different conditions for growing due to their fastidious laboratory growth requirements in comparison to other Gram-negative organisms. These factors suggest that other *Campylobacter* spp. besides *C. jejuni* and *C. coli* are likely underestimated as diarrhea etiologic agents as a result of inappropriate detection and isolation procedures which generally support only the growth of *C. jejuni* and *C. coli* [11].

*Campylobacter concisus* historically has been associated as being a human oral pathogen causing gingivitis and peridontitis [12]. More recent studies have shown that *C. concisus* has been isolated from chronic, more milder diarrheal cases in both adults and children as compared to *C. jejuni* [13–16]. Additionally, *C. concisus* has been implicated in the development of Crohn’s disease and inflammatory bowel disease (IBD) as it has been isolated from fecal samples and colonic biopsies of children with Crohn’s disease and from ulcerative colitis biopsy samples from adults with IBD [17–19]. *C. ureolyticus*, like *C. concisus*, has been detected more frequently in diarrheal cases. A recent study from Hatanaka et al. showed that *C. ureolyticus* was detected in 51.9% of diarrheal stool samples from children with gastroenteritis [20]. Similar studies conducted in Ireland and Chile have also implicated *C. ureolyticus* as an emerging gastrointestinal pathogen [21–23]. Like *C. concisus, C. ureolyticus* has been isolated at high rates from patients with ulcertative colitis and Crohn’s Disease [19, 24].

Currently, there are no to limited data on the prevalence of non-*C. jejuni/C. coli Campylobacter* spp. specifically, *C. ureolyticus* and *C. concisus*, in diarrhea cases in Thailand and Nepal particularly among travelers. In the present/current study, specific pathogen-free stool samples from travelers seeking medical care for traveler’s diarrhea and asymptomatic controls in Nepal and Thailand were assayed for the detection of *C. ureolyticus* and *C. concisus* using *Campylobacter* genus and species specific primers for *C. ureolyticus* and *C. concisus*. To our knowledge, this is the first report of *C. ureolyticus* and *C. concisus* in stool samples in travelers with traveler’s diarrhea and asymptomatic controls from Nepal and Thailand.

## Methods

### Sources of samples

Stool samples used in this study were from two separate epidemiological surveillance studies of TD in Thailand and Nepal conducted in 2011–2014 respectively. The period of enrollment date for the Thailand study was 20 January 2012 to 23 December 2014 and the period of enrollment for the Nepal study was 7 November 2012 to 6 November 2014. For both studies, subjects were residents of North America, Western Europe, Australia, New Zealand, Japan, Taiwan, or South Korea who were 18 years of old or older who visited the Out-patient Department (OPD) or In-patient Department (IPD) of Bumrungrad International Hospital located in Bangkok, Thailand or the CIWEC Clinic Travel Medicine Center in Kathmandu, Nepal. Cases were defined as those individuals with three or more unformed stools per 24 h with at least one additional symptom (nausea, vomiting, abdominal cramps, fecal urgency, tenesmus and fever).

Age-matched controls were residents of North America, Western Europe, Australia, New Zealand, Japan, Taiwan, or South Korea who were 18 years of old or older who visited the Out-patient Department (OPD) or In-patient Department (IPD) of Bumrungrad International Hospital located in Bangkok, Thailand or the CIWEC Clinic Travel Medicine Center in Kathmandu, Nepal. Who had no history of diarrhea in the 2 weeks prior to visiting Bamrungrad International Hospital and the CIWEC Clinic Travel Medicine Center. Subjects who had traveled outside of developed countries or had resided outside of their home countries for more than 30 or 90 days, respectively, within the past year were not enrolled. Controls were recruited by the study team after a diarrheal case was enrolled at Bamrungrad International Hospital and the CIWEC Clinic Travel Medicine Center. After informed consent was obtained, a stool sample was provided by the study volunteer. The Thai study was approved by the Bumrungrad International Institutional Review Board (IRB), Thailand and the Walter Reed Army Institute of Research (WRAIR) IRB, Silver Spring, MD, USA. The Nepal study was approved by the Nepal Health Research Council, Kathmandu, Nepal and the WRAIR IRB. A total of 214 pathogen-negative stool samples (Thailand: 26 cases and 30 controls; Nepal: 83 cases and 75 controls) were used in the study that had previously undergone laboratory testing for enteric bacteria, viruses and parasites as previously described [25, 26]. After testing, pathogen- negative stool samples were archived at −70 °C and stored in Bangkok, Thailand.

### Identification of *C. concisus* and *C. ureolyticus*

DNA was extracted from “pathogen negative” stool samples using the QIAamp Fast DNA Stool Mini Kit (Qiagen, Germantown, MD, USA) according to the manufacturer’s instructions. To identify *Campylobacter* genus positive stool samples, PCR was used to amplify an 816 bp product of *Campylobacter* 16S *rRNA* as previously described using the primers C412F (5′-GGA TGA CAC TTT TCG GAG C-3′) and C1288R (5′-CAT TGT AGC ACG TGT GTC-3′) [27, 28]. *C. ureolyticus* and *C. concisus* were differentiated via PCR using primers for species-specific heat shock protein genes, *Hsp60* and *Cpn60* as previously described [22, 29].

To identify *C. ureolyticus*, the following primers were used to amplify a 429 bp fragment of the heat-shock protein, *Hsp60*: CU-*Hsp60*_F (5′-GAA GTA AAA AGA GGA ATG GAT AAA GAA GC-3′) and CU-*Hsp60*_R (5′-CTT CAC CTT CAA TAT CCT CAG CAA TAA TTA AAA GA-3′) [22]. To identify *C. concisus*, the following primers were used to amplify a 158 bp fragment of the heat shock protein, *cpn60*: JH0023 (5′-GGC TCA AAA AGA GAT CGC TCA-3′) and JH0024 (5′-CCC TCA ACA ACG CTT AGC TC-3′) [29]. The following cycling conditions were used: initial denaturing at 94 °C for 5 min, followed by 40 cycles of 1 min at 94 °C, 1 min at 61 °C for *C. ureolyticus* and 64.6 °C for *C. concisus*, and 1 min at 72 °C and a final extension at 72 °C for 10 min. Positive samples included *C. ureolyticus* American Type Culture Collection (ATCC) 43605 and *C. concisus* ATCC 33237. Negative controls included *Salmonella typhimurium* ATCC 14028, *Shigella flexneri* ATCC 12022, *Shigella sonnei* ATCC 25931, *Vibrio cholera* N16961, enterotoxigenic *Escherichia coli* ATCC 35401, enteropathogenic *E. coli* ATCC 43887, enteroinvasive *E. coli* ATCC 43893 and *Arcobacter butzleri* (laboratory strain). Gels were stained with ethidium bromide and amplicons were visualized using gel electrophoresis. The amplified *C. concisus* (158 bp) and *C. ureolyticus* (429 bp) fragments were sequenced and sequences were confirmed using the Blast Alignment Tool in the National Center for Biotechnology Information database (https://blast.ncbi.nlm.nih.gov/Blast.cgi).

### Statistical analyses

Odds ratios and 95% confidence intervals were calculated using multivariate logistic regression for statistical analysis on case–control data. Data were analyzed using GraphPad Prism Version.

7 (GraphPad Software Inc., La Jolla, CA, USA). A *P* value of <0.05 was considered statistically significant.

## Results

A total of 61 previously determined to be pathogen-negative TD stool samples (CIWEC Travel Medicine Clinic, Nepal: cases = 36, control = 14; Bamrungrad International Hospital, Thailand: cases = 9, controls = 2) were positive for *Campylobacter* 16S *rRNA* (Table 1). Eighteen isolates (29.5%; 18/61) were positive only for *Campylobacter* genus and were not further speciated beyond *C. concisus* and *C. ureolyticus.*

**Table 1:** *C. concisus* and *C. ureolyticus* detected as monoinfections and in mixed infections by PCR using species specific housekeeping genes in pathogen-negative traveler’s diarrhea cases and asymptomatic controls from CIWEC Travel Medicine Clinic, Kathmandu, Nepal and Bamrungrad International Hospital, Bangkok, Thailand

CIWEC Travel Medicine Clinic, Nepal	Cases (n = 83)	Controls (n = 75)	OR (95% CI)	*P*
*C. concisus*: single pathogen	24 (28.9%)	3 (4%)	9.8 (2.8–34.1)	0.0003
*C. ureolyticus:* single pathogen	4 (4.8%)	4 (5.3%)	0.9 (0.2–3.7)	NS
*C. concisus* and *C. ureolyticus* mixed infection	2 (2.4%)	1 (1.3%)	1.8 (0.2–20.5)	NS
*Campylobacter* spp. *(*non *C. concisus and C. ureolyticus*)	6 (7.2%)	6 (8.0%)	0.7 (0.2–2.0)	NS

Bamrungrad International Hospital, Thailand	Cases (n = 26)	Controls (n = 30)	OR (95% CI)	*P*
*C. concisus*: Single pathogen	2 (7.7%)	0	N/A	N/A
*C. ureolyticus:* Single pathogen	1 (3.8%)	1 (3.3%)	1.2 (0.07–19.5)	NS
*C. concisus* and *C. ureolyticus* mixed infection	1 (3.8%)	0	N/A	N/A
*Campylobacter* spp. (non *C. concisus and C. ureolyticus*)	5 (19.2%)	1 (3.3%)	4.8 (0.5–41.6)	NS

Odds ratios (OR) and 95% confidence intervals (95% CI) were calculated using multivariate logistic regression with a significant *P* < 0.05

*NS* not significant

*N/A* not applicable for statistical analysis

At the CIWEC Travel Medicine Clinic, Nepal, *C. consisus* was identified significantly more often in TD cases (28.9%; 24/83) when compared to asymptomatic controls (4%; 3/75) (OR = 9.8; 95% CI 2.8–34.1; *P* = 0.0003) when isolated as the sole pathogen (Table 1). *C. ureolyticus* was detected in four cases (4.8%; 4/83) and four controls (5.3%; 4/75) respectively (OR = 0.90; 95% CI 0.2–3.7; *P* = 0.88) when isolated as the sole pathogen.

*Campylobacter concisus* and *C. ureolyticus* mixed infections were detected in two cases (2.4%; 2/83) and in one control (1.3%; 1/75) sample (OR = 1.8; 95% CI 0.2–20.5; *P* = 0.62).

At the Bamrungrad International Hospital, Thailand, *C. consisus* was identified in only two TD cases (7.7%; 2/26) and in none of the controls (0%; 0/30) (Table 1). *C. ureolyticus* was detected in one case (3.8%; 1/26) and in one control (3.1%; 1/30) (OR = 1.16; 95% CI 0.07–19.5; *P* = 0.92) in the stool samples collected at Bamrungrad. *C. concisus* and *C. ureolyticus* as a mixed infection were detected in one case (3.8%; 1/26) and in none of the control samples.

In Table 2, the number of *C. jejuni* and *C. coli* isolates detected by culture are listed in both monoinfections and mixed infections by site. *C. jejuni* was detected significantly more often in the cases (7.9%; 38/480) when compared to the controls (2.9%; 6/209) at the CIWEC Travel Medicine Clinic, Nepal where *C. jejuni* was the sole pathogen isolated (OR = 6.5; 95% CI 2.2–19.1; *P* = 0.0007). *C. coli* was detected slightly more often in the cases (1.5%; 7/480) when compared to the controls (1.4%; 3/209) though not significantly (OR = 1.0; 95% CI 0.3–3.9; *P* = 0.9) when *C. coli* was the sole pathogen detected. In mixed infections, *C. jejuni* was detected significantly more often in the cases (8.1%; 39/480) when compared to the controls (1.9%; 4/209) (OR = 20.2; 95% CI 2.7–152.9; *P* = 0.0036) (Table 2). *C. coli* was detected more often in the cases (1.3%; 6/480) when compared to the controls (0.9%; 2/209) though not significantly in mixed infections (OR = 1.3; 95% CI 0.3–6.6; *P* = 0.74) (Table 2).

**Table 2:** *C. jejuni* and *C. coli* isolated as single pathogens and from mixed infections via culture in traveler’s diarrhea cases and asymptomatic controls from CIWEC Travel Medicine Clinic, Kathmandu, Nepal and Bamrungrad International Hospital, Bangkok, Thailand

CIWEC Travel Medicine Clinic, Nepal	Cases (n = 480)	Controls (n = 209)	OR (95% CI)	*P*
*C. jejuni:* single pathogen	38 (7.9%)	6 (2.9%)	6.5 (2.2–19.1)	0.0007
*C. coli:* single pathogen	7 (1.5%)	3 (1.4%)	1.0 (0.3–3.9)	NS
*C. jejuni*: mixed infection	39 (8.1%)	4 (1.9%)	20.2 (2.7–152.9)	0.0036
*C. coli*: mixed infection	6 (1.3%)	2 (0.9%)	1.3 (0.3–6.6)	NS

Bamrungrad International Hospital, Thailand	Cases (n = 173)	Controls (n = 165)	OR (95% CI)	*P*
*C. jejuni:* single pathogen	24 (13.9%)	4 (2.4%)	2.9 (1.2–6.9)	0.01
*C. coli:* single pathogen	4 (2.3%)	0	N/A	N/A
*C. jejuni*: mixed infection	19 (11%)	1 (0.6)	20.2 (2.7–152.9)	0.0036
*C. coli*: mixed infection	5 (2.9%)	0	N/A	N/A

Odds ratios (OR) and 95% confidence intervals (95% CI) were calculated using multivariate logistic regression with a significant *P* < 0.05

*NS* not significant

*N*/*A* not applicable for statistical analysis

At Bamrungrad International Hospital, Thailand, *C. jejuni* was detected significantly more often in the cases (13.9%; 24/173) when compared to the controls (2.4%; 4/165) when *C. jejuni* was the sole pathogen isolated (OR = 2.9; 95% CI 1.2–6.9; *P* = 0.01) (Table 2). *C. coli* was detected only in the cases (2.3%; 4/173) when *C. coli* was isolated as a single pathogen. *C. jejuni* was detected significantly more often in the cases (11%; 19/173) when compared to the controls (0.6%; 1/165) (OR = 20.2; 95% CI 2.7–152.9; *P* = 0.0036) in mixed infections. In mixed infections, *C. coli* was detected only in the cases (2.9%; 5/173) at Bamrungrad International Hospital, Thailand.

## Discussion

The prevalence of *Campylobacter* spp. as etiologic agents for TD in Nepal and Thailand has been well documented, but the vast majority of these studies have focused on the detection of *C. jejuni* and *C. coli* as the primary *Campylobacter* spp [4, 5, 30–32]. More recently, other *Campylobacter* spp. to include *C. concisus* and *C. ureolyticus* have been implicated in human gastroenteritis due to improved methods of laboratory detection. Most clinical laboratories do not regularly test for other *Campylobacter* spp. such as *C. concisus* and *C. ureolyticus* due to both the fastidious nature of the organisms, the requirement for an enriched H_2_ environment in order for growth and both species being unable to grow on the selective media that is commonly used for *C. jejuni* and *C. coli* [16, 20, 33]. It is highly probable that due to these reasons, gastroenteritis caused by *C. concisus* and *C. ureolyticus* is highly underestimated. In TD etiologic studies, *C. concisus* and *C. ureolyticus* are rarely tested for and therefore the incidence of *C. concisus* and *C. ureolyticus* in TD is largely unknown.

In this study, *C. concisus* was detected significantly more often in TD stool samples from Nepal as compared to asymptomatic controls. These stools samples were previously categorized as pathogen-negative after undergoing a comprehensive laboratory workup for enteric bacteria, viruses and parasites. In the present study, 92.3% of the *C. concisus* cases were from Nepal. Similarly, 80% of the *C. ureolyticus* cases were from Nepal though there were only five total cases of *C. ureolyticus* detected. There are no previous published data on the prevalence of *C. concisus* and *C. ureolyticus* from the CIWEC Travel Medicine Clinic, Kathmandu, Nepal and at Bamrungrad International Hospital, Bangkok, Thailand as these microorganisms are not routinely tested for in the clinical laboratories at these facilities. In a previous TD case–control study conducted at the CIWEC Travel Medicine Clinic from 2001 to 2003, *Campylobacter* was the most prevalent pathogen detected in cases (17%), but the *Campylobacter* isolates were not speciated in this study [4]. Multiple studies have also shown that *Campylobacter* spp. is the most common pathogen in Thailand to cause acute gastroenteritis in travelers, deployed US military personnel and the indigenous population [5, 6, 31, 34, 35]. Similar to cases from CIWEC, *C. jejuni* was the most common detected pathogen from cases at Bamrungrad International Hospital. To our knowledge, this is the first report showing detection of *C. concisus* and *C. ureolyticus* in TD samples in a case–control setting from Nepal and Thailand and that these results strongly suggest a potential role of *C. concisus* in the development of TD in travelers to Nepal.

Recent studies have shown that the prevalence of *C. concisus* and *C. ureolyticus* typically are equal or higher when compared to *C. jejuni* and *C. coli* in studies in which they are both included in the laboratory workup [36–38]. In Australia, Underwood et al. showed that *C. concisus* had a prevalence of 49.7% in patients with gastroenteritis as compared to *C. jejuni* (5%) and that it was detected as a single agent in 78% of the *C. concisus* positive cases indicating a potential role for *C. concisus* in the development of gastroenteritis in these patients [16]. In a 2 year study conducted in Denmark, Nielsen et al. showed that *C. concisus* was prevalent in 26.1% of patients with gastroenteritis as compared to *C. jejuni/coli* detected in 31.9% of infected patients [15]. In the first report of *C. ureolyticus* in children with gastroenteritis in Japan, Hatanaka et al. showed that *C. ureolyticus* was prevalent in 51.9% of the infected children as compared to *C. jejuni/coli* infecting 15.5% of the children [20]. In a study from Southern Ireland, *C. ureolyticus* was prevalent in 41% of diarrheal patients as compared to *C. jejuni* at 50.7% and *C. coli* at 5.7% [28]. In the present study, only the incidence of *C. concisus* in the Nepal pathogen-negative TD cases (28.9%) was higher than the incidence of both *C. jejuni* (16.0%) and *C. coli* (2.7%) in the Nepal TD cases that yielded at least one enteric pathogen under routine laboratory testing. It is likely that the incidence of *C. concisus* and *C. ureolyticus* are highly underestimated due to the fact that detection of these organisms was not included in the initial routine laboratory testing of the TD cases from both CIWEC and Bamrungrad hospital.

There were several limitations to the study. As mentioned previously, only pathogen-negative stools were selected which limited the number of samples used. Between the two sites, there were 653 cases and 374 controls (total = 1027). This resulted in a smaller sample size particularly for the TD samples from Thailand (26 cases and 30 controls) as well as an underestimation of the overall prevalence of both *C. concisus* and *C. ureolyticus* in all of the cases from both sites. The selection of pathogen-negative stools also inhibited our ability to address co-infections with *C. concisus* and *C. ureolyticus.* However, since pathogen-negative samples were solely used, we can infer from the data that those samples containing *C. concisus* or *C. ureolyticus* were monoinfections with no other enteric pathogens being detected previously. As both organisms have been implicated in the development of gastroenteritis, there have been recent studies to include whole genome sequencing aimed at delineating virulence factors to elucidate the pathogenic mechanisms of each organism. Both *C. concisus* and *C. ureolyticus* contain genes similar to *C. jejuni* for adhesins, invasins and toxins that are known to play an important role in the development of acute gastroenteritis [37, 39–42]. These virulence factors were not tested for in the present study. Additionally, PCR assays should have been included for the detection of *C. jejuni* and *C. coli* as PCR has shown to be far superior for the detection of both *C. jejuni* and *C. coli* when compared to culture [43–46]. It is likely that the overall estimate of *C. jejuni* and *C. coli* by PCR would increase to those that were positive by culture. Future plans include testing all 826 samples using PCR for *C. jejuni, C. coli, C. concisus* and *C. ureolyticus* to better compare the prevalence of each organism using a similar molecular method of detection and in the same batch of samples.

As this is the first report of *C. concisus* and *C. ureolyticus* in acute gastroenteritis in travelers to Nepal and Thailand, further studies with long term clinical follow up of infected travelers should be initiated to assess the development of any long term post infectious sequelae to include IBD and ulcerative colitis that have been observed in studies examining the long term effects of *Campylobacter* infection [47, 48]. In conclusion, as it is evident that at least *C. concisus* from this study is involved in the development of acute gastroenteritis in travelers to Nepal, it is imperative that more studies include both *C. concisus* and *C. ureolyticus* are included in the laboratory diagnostic methods to better ascertain their true prevalence as etiological agents of acute gastroenteritis. Additionally, there are limited data on the geographic distribution of *C. concisus* and *C. ureolyticus* in developing and developed countries and further epidemiological studies to ascertain this are warranted.

## Conclusions

The prevalence of *C. ureolyticus* and *C. concisus* in traveler’s diarrhea cases in travelers to Nepal and Thailand largely unknown. In this study, pathogen-negative stool samples from travelers seeking medical care for traveler’s diarrhea and asymptomatic controls in Nepal and Thailand were assayed by PCR for the detection of *C. ureolyticus* and *C. concisus* using *Campylobacter* genus and species specific primers for *C. ureolyticus* and *C. concisus*. The results of the study show that *C. concisus* is potentially involved in the development of acute gastroenteritis in travelers to Nepal as it was detected significantly more often in cases when compared to controls. Future traveler’s diarrhea studies should include *C. concisus* and *C. ureolyticus* in their laboratory diagnostics menu to better estimate their potential as etiological agents of traveler’s diarrhea.

## References

[1] Whiley H, van den Akker B, Giglio S, Bentham R (2013). The role of environmental reservoirs in human campylobacteriosis. Int J Environ Res Public Health..

[2] Kaakoush NO, Castano-Rodriguez N, Mitchell HM, Man SM (2015). Global epidemiology of *Campylobacter* infection. Clin Microbiol Rev.

[3] Shah N, DuPont HL, Ramsey DJ (2009). Global etiology of travelers’ diarrhea: systematic review from 1973 to the present. Am J Trop Med Hyg.

[4] Pandey P, Bodhidatta L, Lewis M (2011). Travelers’ diarrhea in Nepal: an update on the pathogens and antibiotic resistance. J Travel Med..

[5] Serichantalergs O, Pootong P, Dalsgaard A (2010). PFGE, Lior serotype, and antimicrobial resistance patterns among *Campylobacter jejuni* isolated from travelers and US military personnel with acute diarrhea in Thailand, 1998–2003. Gut Pathog..

[6] Jiang ZD, DuPont HL (2017). Etiology of travellers’ diarrhea. J Travel Med..

[7] Fernandez H, Vera F, Villanueva MP, Garcia A (2008). Occurrence of *Campylobacter* species in healthy well-nourished and malnourished children. Braz J Microbiol..

[8] Man SM (2011). The clinical importance of emerging *Campylobacter* species. Nat Rev Gastroenterol Hepatol..

[9] Taylor DN, Kiehlbauch JA, Tee W, Pitarangsi C, Echeverria P (1991). Isolation of group 2 aerotolerant *Campylobacter* species from Thai children with diarrhea. J Infect Dis.

[10] Lastovica AJ, le Roux E (2000). Efficient isolation of *Campylobacteria* from stools. J Clin Microbiol.

[11] Lastovica AJ (2006). Emerging *Campylobacter* spp.: the tip of the iceberg. Clin Microbiol Newsl..

[12] Zhang L, Budiman V, Day AS (2010). Isolation and detection of *Campylobacter concisus* from saliva of healthy individuals and patients with inflammatory bowel disease. J Clin Microbiol.

[13] Nielsen HL, Engberg J, Ejlertsen T, Bucker R, Nielsen H (2012). Short-term and medium-term clinical outcomes of *Campylobacter concisus* infection. Clin Microbiol Infect.

[14] Nielsen HL, Engberg J, Ejlertsen T, Nielsen H (2013). Clinical manifestations of *Campylobacter concisus* infection in children. Pediatr Infect Dis J..

[15] Nielsen HL, Ejlertsen T, Engberg J, Nielsen H (2013). High incidence of *Campylobacter* concisus in gastroenteritis in North Jutland, Denmark: a population-based study. Clin Microbiol Infect.

[16] Underwood AP, Kaakoush NO, Sodhi N (2016). *Campylobacter concisus* pathotypes are present at significant levels in patients with gastroenteritis. J Med Microbiol.

[17] Engberg J, On SL, Harrington CS, Gerner-Smidt P (2000). Prevalence of *Campylobacter*, *Arcobacter*, *Helicobacter*, and *Sutterella* spp. in human fecal samples as estimated by a reevaluation of isolation methods for *Campylobacters*. J Clin Microbiol.

[18] Van Etterijck R, Breynaert J, Revets H (1996). Isolation of *Campylobacter concisus* from feces of children with and without diarrhea. J Clin Microbiol.

[19] Man SM, Zhang L, Day AS, Leach ST, Lemberg DA, Mitchell H (2010). C*ampylobacter concisus* and other *Campylobacter* species in children with newly diagnosed Crohn’s disease. Inflamm Bowel Dis.

[20] Hatanaka N, Shimizu A, Somroop S (2017). High prevalence of Campylobacter ureolyticus in stool specimens of children with diarrhea in Japan. Jpn J Infect Dis.

[21] Bullman S, Corcoran D, O’Leary J, O’Hare D, Lucey B, Sleator RD (2011). Emerging dynamics of human campylobacteriosis in Southern Ireland. FEMS Immuno Med Microbiol..

[22] Bullman S, Corcoran D, O’Leary J, Lucey B, Byrne D, Sleator RD (2011). *Campylobacter ureolyticus*: an emerging gastrointestinal pathogen?. FEMS Immunol Med Microbiol.

[23] Collado L, Gutierrez M, Gonzalez M, Fernandez H (2013). Assessment of the prevalence and diversity of emergent campylobacteria in human stool samples using a combination of traditional and molecular methods. Diagn Microbiol Infect Dis.

[24] Mukhopadhya I, Thomson JM, Hansen R, Berry SH, El-Omar EM, Hold GL (2011). Detection of *Campylobacter concisus* and other *Campylobacter* species in colonic biopsies from adults with ulcerative colitis. PLoS ONE.

[25] Bodhidatta L, McDaniel P, Sornsakrin S, Srijan A, Serichantalergs O, Mason CJ (2010). Case-control study of diarrheal disease etiology in a remote rural area in Western Thailand. Am J Trop Med Hyg.

[26] Meng CY, Smith BL, Bodhidatta L (2011). Etiology of diarrhea in young children and patterns of antibiotic resistance in Cambodia. Pediatr Infect Dis J..

[27] Linton D, Owen RJ, Stanley J (1996). Rapid identification by PCR of the genus *Campylobacter* and of five *Campylobacter* species enteropathogenic for man and animals. Res Microbiol.

[28] Bullman S, O’Leary J, Corcoran D, Sleator RD, Lucey B (2012). Molecular-based detection of non-culturable and emerging *campylobacteria* in patients presenting with gastroenteritis. Epidemiol Infect.

[29] Chaban B, Musil KM, Himsworth CG, Hill JE (2009). Development of cpn60-based real-time quantitative PCR assays for the detection of 14 *Campylobacter* species and application to screening of canine fecal samples. Appl Environ Microbiol.

[30] Murphy H, Pandey P (2012). Pathogens for travelers’ diarrhea in Nepal and resistance patterns. Curr Infect Dis Rep..

[31] Taylor DN, Echeverria P, Pitarangsi C, Seriwatana J, Bodhidatta L, Blaser MJ (1988). Influence of strain characteristics and immunity on the epidemiology of *Campylobacter* infections in Thailand. J Clin Microbiol.

[32] Walz SE, Baqar S, Beecham HJ (2001). Pre-exposure anti-*Campylobacter jejuni* immunoglobulin a levels associated with reduced risk of *Campylobacter* diarrhea in adults traveling to Thailand. Am J Trop Med Hyg.

[33] Vandamme P, Debruyne L, De Brandt E, Falsen E (2010). Reclassification of *Bacteroides ureolyticus* as *Campylobacter ureolyticus* comb. nov., and emended description of the genus *Campylobacter*. Int J Syst Evol Microbiol.

[34] Varavithya W, Vathanophas K, Bodhidatta L (1990). Importance of *Salmonellae* and *Campylobacter jejuni* in the etiology of diarrheal disease among children less than 5 years of age in a community in Bangkok, Thailand. J Clin Microbiol..

[35] Serichantalergs O, Dalsgaard A, Bodhidatta L (2007). Emerging fluoroquinolone and macrolide resistance of *Campylobacter jejuni* and *Campylobacter coli* isolates and their serotypes in Thai children from 1991 to 2000. Epidemiol Infect.

[36] Kaakoush NO, Mitchell HM (2012). *Campylobacter concisus*—a new player in intestinal disease. Front Cell Infect Microbiol..

[37] Burgos-Portugal JA, Kaakoush NO, Raftery MJ, Mitchell HM (2012). Pathogenic potential of *Campylobacter ureolyticus*. Infect Immun.

[38] Lee S, Lee J, Ha J (2016). Clinical relevance of infections with zoonotic and human oral species of *Campylobacter*. J Microbiol..

[39] Kalischuk LD, Inglis GD (2011). Comparative genotypic and pathogenic examination of *Campylobacter concisus* isolates from diarrheic and non-diarrheic humans. BMC Microbiol.

[40] Kaakoush NO, Man SM, Lamb S (2010). The secretome of *Campylobacter concisus*. FEBS J.

[41] Man SM, Kaakoush NO, Leach ST (2010). Host attachment, invasion, and stimulation of proinflammatory cytokines by Campylobacter concisus and other non-*Campylobacter jejuni Campylobacter* species. J Infect Dis.

[42] Bullman S, Lucid A, Corcoran D, Sleator RD, Lucey B (2013). Genomic investigation into strain heterogeneity and pathogenic potential of the emerging gastrointestinal pathogen *Campylobacter ureolyticus*. PLoS ONE.

[43] Buchan BW, Olson WJ, Pezewski M (2013). Clinical evaluation of a real-time PCR assay for identification of *Salmonella*, *Shigella*, *Campylobacter* (*Campylobacter jejuni* and *C. coli*), and shiga toxin-producing *Escherichia coli* isolates in stool specimens. J Clin Microbiol.

[44] Schuurman T, de Boer RF, van Zanten E (2007). Feasibility of a molecular screening method for detection of *Salmonella enterica* and *Campylobacter jejuni* in a routine community-based clinical microbiology laboratory. J Clin Microbiol.

[45] Wohlwend N, Tiermann S, Risch L, Risch M, Bodmer T (2016). Evaluation of a multiplex real-time PCR assay for detecting major bacterial enteric pathogens in fecal specimens: intestinal inflammation and bacterial load are correlated in *Campylobacter* infections. J Clin Microbiol.

[46] Platts-Mills JA, Liu J, Gratz J (2014). Detection of *Campylobacter* in stool and determination of significance by culture, enzyme immunoassay, and PCR in developing countries. J Clin Microbiol.

[47] Gradel KO, Nielsen HL, Schonheyder HC, Ejlertsen T, Kristensen B, Nielsen H (2009). Increased short- and long-term risk of inflammatory bowel disease after *Salmonella* or *Campylobacter* gastroenteritis. Gastroenterology.

[48] Riddle MS, Gutierrez RL, Verdu EF, Porter CK (2012). The chronic gastrointestinal consequences associated with *Campylobacter*. Curr Gastroenterol Rep.

